# Health Effects and Therapeutic Potential of the Gut Microbe *Akkermansia muciniphila*

**DOI:** 10.3390/nu17030562

**Published:** 2025-01-31

**Authors:** Ezinne Aja, Amber Zeng, Weston Gray, Kaden Connelley, Anil Chaganti, Jonathan P. Jacobs

**Affiliations:** 1 Goodman-Luskin Microbiome Center, University of California, Los Angeles, CA 90095, USA; eaja@mednet.ucla.edu; 2 UCLA Vatche and Tamar Manoukian Division of Digestive Diseases, Department of Medicine, David Geffen School of Medicine, Los Angeles, CA 90095, USA; amberzeng@ucla.edu (A.Z.); weston.gray@tufts.edu (W.G.); kadenkc@g.ucla.edu (K.C.); anilchaganti30@gmail.com (A.C.); 3 Division of Gastroenterology, Hepatology and Parenteral Nutrition, Veterans Affairs Greater Los Angeles Healthcare System, Los Angeles, CA 90073, USA

**Keywords:** *Akkermansia muciniphila*, probiotic, gut microbiota, obesity, diabetes, intestinal barrier, mucin, short-chain fatty acids

## Abstract

*Akkermansia muciniphila* is a bacterium commonly found in the human gastrointestinal tract that has received considerable interest as a potential probiotic for the improvement of gut health and overall metabolic function. *A. muciniphila* is enriched in the mucus layer of the intestinal lining, where it degrades mucin and plays a significant role in gut barrier maintenance and immune regulation. A higher abundance of *A. muciniphila* has been observed in the gut of healthy individuals relative to those with metabolic disorders, and multiple metabolic benefits, including improved glucose management, reduced body fat, and reduced inflammation have been linked to *A. muciniphila*. Current research on *A. muciniphila* primarily relies on mouse models, with limited human interventional studies available. While these animal studies offer valuable insights into the potential roles of *A. muciniphila* in health and disease, further clinical investigations in humans are needed to fully understand its impact. Here, we explore the current scope of *A. muciniphila* research and its potential as a therapeutic agent to improve gut and metabolic health while also emphasizing the need to optimize techniques to further improve studies of this organism.

## 1. Introduction

The gastrointestinal (GI) tract has a diverse microbial ecology that greatly influences host health and physiology [1]. The host-derived mucus layer that separates the microbial community in the lumen of the GI tract from intestinal epithelial cells is inhabited by microorganisms. One such microbe, *Akkermansia muciniphila*, has received significant attention in recent years. This unique bacterium, first isolated in 2004 [2], has emerged as a potential key player in maintaining human health and preventing various disease states. Studies have demonstrated that *A. muciniphila* can protect against pathogens and reduce inflammation by promoting intestinal barrier function. *A. muciniphila* has also been associated with the prevention of obesity, including reduced adiposity and increased insulin sensitivity [3,4]. This is associated with the altered metabolism of fatty acids and bile acids, potentially impacting host metabolism [5,6]. In this review, the current state of *A. muciniphila* research is discussed, highlighting the recent advances in investigating its mechanisms of action, its significance in human health, and its prospective therapeutic applications.

## 2. Genetic and Metabolic Properties of *A. muciniphila*

### 2.1. Taxonomy and Genetics

*A. muciniphila* belongs to the phylum *Verrucomicrobia* and was the first cultivated member of the *Akkermansia* genus [7]. It is a Gram-negative, oval-shaped, non-spore-forming, non-motile, oxygen-tolerant anaerobic bacterium that was discovered in 2004 in a fecal sample from a healthy human volunteer. *A. muciniphila* was named to honor Antoon DL Akkermans and highlight the ability of this new bacterial species to degrade mucin and efficiently exploit mucus as its principal source of carbon and nitrogen [2,8,9,10]. Studies have shown that while *A. muciniphila* is abundantly found in the colon, it is also found in the mucus layer of the small intestine [11]. In 2011, the genome of *A. muciniphila* was sequenced, demonstrating its specialization in mucin breakdown and its probable function at the mucosal interface between the lumen and host cells. The 2.7 Mb genome was predicted to encode 61 proteins involved in mucin degradation (2.8% of all predicted proteins), including several glycosyl hydrolases, proteases, sulfatases, and sialidases [12]. The analysis also noted that 43% of all proteins predicted to be secreted were annotated as hypothetical proteins, many of which may be involved in mucin degradation and processing. Passel and others conducted a comprehensive genomic analysis of *A. muciniphila* that showed the existence of two CRISPR loci and many phage-derived sequences in the genome, indicating that viral infections have significantly influenced the evolutionary history and speciation of *A. muciniphila* [12,13].

While the Type strain of *A. muciniphila*, MucT (ATCC BAA-835), has been extensively studied, recent studies have indicated that there are numerous strains of *A. muciniphila* that have significant genomic and phenotypic diversity [14]. A thorough genomic investigation indicated the presence of 215 strains and 234 isolates of *A. muciniphila* available in the NCBI database [6]. These strains could be classified into separate clades according to their 16S rRNA sequences, suggesting that *A. muciniphila* encompasses a complex group of strains, potentially with subspecies with diverse attributes. These strains show notable differences in their gene content, which may affect their functional capacities and interactions within the gut microbiome [15]. For example, *A. muciniphila* strains vary in generation of short-chain fatty acids (SCFAs), which are crucial for gastrointestinal health and metabolic processes [13]. Strains also vary greatly in their tolerance to oxygen, an important property for colonizing the intestinal epithelial surface [13]. Other studies have suggested differences among strains of *A. muciniphila* in their effects on *Clostridioides difficile* and intestinal inflammation [16,17]. It has recently been suggested that some isolates reported as strains of *A. muciniphila* may be distinct enough to comprise new species of *Akkermansia* [18].

### 2.2. Oxygen Tolerance

Recent studies have disputed the rigid classification of *A. muciniphila* as anaerobic. Evidence suggests that *A. muciniphila* may tolerate short-term exposure to oxygen and exhibit some properties of facultative anaerobes [19,20]. Findings by Becken and colleagues provide evidence for greater adaptability of *A. muciniphila* than previously seen [13]. Their studies showed that *A. muciniphila* strains may thrive in low-oxygen environments and maintain significant survival rates under microaerophilic conditions representative of oxygen levels that may be encountered near the mucosal surface of the GI tract.

### 2.3. Metabolism and Mucus Degradation

As *A. muciniphila* is known to colonize the host’s intestinal mucosa [21], it is unsurprising that many of its energy acquisition pathways center around mucin degradation and processing. *A. muciniphila* is predominantly distributed in the distal regions of the small and large intestines, where it primarily utilizes mucin as an energy source to synthesize the amino acids and sugar groups essential for bacterial growth. A study using a genome-scale metabolic model and in vitro validation found that *A. muciniphila* can utilize the mucin-derived monosaccharides fucose, galactose, *N*-acetylglucosamine (GlcNAc), and *N*-acetyl galactosamine (GalNAc) as its primary carbon and nitrogen sources [22]. Growth rates on single sugars were notably lower—or in the case of galactose, did not occur—compared to growth rates on mucin; yet, in all cases, a mixture of mucin and a single sugar increased the growth rate, indicating *A. muciniphila*’s reliance on cometabolism of sugars and need for additional mucin-derived components for efficient growth. *A. muciniphila* is capable of fermenting other non-mucin sugars, including fructose [22] and human milk oligosaccharides [23]. The di- and trisaccharides maltose, melibiose, trehalose, and raffinose have not been observed to be metabolized by *A. muciniphila* [22], although a genome analysis did suggest its ability to metabolize melibiose and cellobiose [12].

Glucose metabolism in *A. muciniphila* is rapid but not sustained, indicating a depletion of some factor that prevents such growth on the monosaccharide. Growth on fucose and a combination of fucose and glucose yielded similar results. Given that growth on the amino sugars GlcNAc and GalNAc both promote sustained, linear growth, it is plausible that the limiting factor in sustained non-amino sugar metabolism is a favorable nitrogen source, which would be supplied by GlcNAc and GalNAc in vivo. Glucose metabolism genes, such as those coding for alpha-amylases and alpha-glucosidases, were found to be upregulated with glucose administration as compared to mucin. However, multiple proteins commonly involved in the bacterial stress response were upregulated in glucose cultures, indicating that the use of glucose as the sole carbon source for *A. muciniphila* is a nonoptimal, stressful environmental condition. These phenomena point to glucose metabolism in *A. muciniphila* as a temporary mechanism of energy acquisition upon displacement into the fiercely competitive luminal environment [22].

*A. muciniphila* is known to produce short-chain fatty acids (SCFAs) as products of sugar catabolism, namely: acetate, propionate, and small amounts of succinate. Succinate, which is usually an intermediate in the conversion of dietary carbohydrates to propionate [24], is excreted outside of the cell upon its accumulation, as the conversion of succinate to propionate appears to be a metabolic bottleneck. Succinate is also notably taken up by *A. muciniphila* during the stationary phase of growth, indicating its potential use in further energy gain, which is supported by the metabolic model. Surprisingly, acetate is not produced from acetyl-CoA via acetate kinase but rather is produced when CoA is transferred to succinate to form succinyl-CoA, an intermediate in the propionate cycle. 1,2-Propanediol is also produced in small amounts from the metabolism of fucose. Some evidence suggests a pathway in *A. muciniphila* for the conversion of 1,2-propanediol to propionate; however, genome-scale metabolic models show that these genes are not present [22]. Vitamin B12 plays an important role in the conversion of succinate to propionate, acting as a cofactor in the reaction. Interestingly, one study found that only one-third of *A. muciniphila* isolates were capable of producing vitamin B12, indicating the necessity of environmental vitamin B12 for the majority of *A. muciniphila* strains [25,26,27].

Of the nine essential amino acids, threonine is the only one that *A. muciniphila* is unable to synthesize on its own [22]. Given that threonine is notably abundant in intestinal mucin [28], this is likely a consequence of adaptation to the mucosal environment.

## 3. Factors Influencing *A. muciniphila* Abundance

### 3.1. Diet and Dietary Components

Diet shapes the structure, composition, and function of the human gut microbiome. The effects of dietary inputs on *A. muciniphila* are discernible almost immediately after birth. A recent study investigated the impact of the breast milk-derived metabolite betaine [29], a trimethylated derivative of glycine, on the overall relative abundance of *A. muciniphila*. Maternal betaine was found to transiently increase *A. muciniphila* abundance in vivo and in vitro. Human milk oligosaccharides [23], unconjugated complex carbohydrates that serve as prebiotics for the neonatal gut microbiota, were also shown to promote the expansion of *A. muciniphila*.

High-fat diet exposure has been reported to significantly deplete *A. muciniphila* in mice [30]. In addition to possessing elevated levels of saturated fats and refined sugars, the Western diet is deficient in prebiotic dietary fiber. Dietary fiber supplementation has been shown to modulate *A. muciniphila* abundance. More specifically, existing studies have shown that supplementation with inulin, butyrate, psyllium, and oat-derived β-glucan, among other common dietary fibers, may lead to an increase in the relative abundance of *A. muciniphila* [31,32,33,34]. There are also multiple studies demonstrating that dietary polyphenols, plant-derived compounds with antioxidant and anti-inflammatory properties, increase the relative abundance of *A. muciniphila* [35]. Roopchand et al. investigated the effects of grape polyphenols, stabilized in a soy protein isolate matrix, on obesity-related metabolic syndrome in mice, with focus on the gut microbiome composition [36]. Following the administration of a high-fat diet supplemented with grape polyphenols, *A. muciniphila* levels increased significantly. It was speculated that grape polyphenols have selective antibacterial effects that target competing microbes, thereby affording *A. muciniphila* the opportunity to expand. While it is unclear whether the relationship between grape polyphenols and the gut microbiota is facilitated by host-derived physiological inputs, a study by Kemperman and colleagues demonstrated that grape polyphenols directly increased the *A. muciniphila* abundance in vitro. Similar effects were observed with polyphenols derived from chokeberry [37].

### 3.2. Antibiotics and Other Medications

Antibiotics, both broad and narrow spectrum, engender widespread shifts in the gut microbiome [38]. Early childhood exposure to antibiotics has been shown to reduce gut microbiota species diversity, promote antibiotic resistance, and lead to a higher incidence of gastrointestinal, immunologic, and neurocognitive disease states. Maier et al. shed light on *A. muciniphila*’s response to broad-spectrum antibiotic administration, highlighting its resistance to a vast majority of quinolone antibiotics [39]. Another study found that the *A. muciniphila* MucT strain is resistant to a handful of antibiotics, including vancomycin and metronidazole [40]. Furthermore, the *Verrucomicrobia* phylum, which contains *A. muciniphila*, has been shown to expand in the human gastrointestinal tract following antibiotic therapy [41]. Given that *A. muciniphila* can acquire genes from neighboring bacteria via lateral gene transfer [42], it may be less susceptible to the effects of broad-spectrum antibiotics.

Non-antibiotic medications have also been shown to modulate gut microbiome composition in both animal and human studies. Metformin (1,1-dimethylbiguanide hydrochloride), a common antihyperglycemic drug used to treat type 2 diabetes mellitus, has been associated with an increased *A. muciniphila* abundance in human and mouse studies [19,43,44]. In addition, metformin can induce the upregulation of metabolic pathways related to lipopolysaccharide biosynthesis, sphingolipid metabolism, and pentose and glucuronate interconversions, demonstrating its potential to modulate *A. muciniphila* function as well as its abundance [45].

There is emerging evidence that herbal medicines, particularly those used in traditional Chinese medicine, impact *A. muciniphila* abundance. A recent study investigated the effect of supplementing a high-fat and high-sucrose diet with an anthraquinone-rich Chinese rhubarb (*Rheum palmatum*) extract in mice. By analyzing microbial shifts at the phylum and genus levels post-supplementation, it was found that Chinese rhubarb significantly increased the relative abundance of *A. muciniphila* [46]. Puerarin, a bioactive isoflavonoid that is commonplace in traditional Chinese food and medicine, was also shown to enrich *A. muciniphila* in obese mice, thereby partially ameliorating the deleterious effects of an HFD [47]. In addition, there is evidence that Bofutsushosan, a Japanese herbal medicine, leads to beneficial restructuring of the gut microbiome, favoring *A. muciniphila* expansion [48,49].

### 3.3. Age-Related Changes

Senescence, or biological aging, produces significant alterations in gut microbiome function and composition. A decline in overall host immune function and the increased prevalence of age-associated diseases may be attributed in part to a weakening of the intestinal mucosal barrier. There is evidence that the abundance of *A. muciniphila* decreases with age [50], with elderly subjects exhibiting a notable reduction in *A. muciniphila* levels when compared to young and middle-aged adults. Conversely, a noticeable expansion of *A. muciniphila* was observed in centenarian subjects [51]. These complementary results allude to the importance of *A. muciniphila* in longevity. In a mouse model of aging, supplementation with *A. muciniphila* induced thickening of the colonic mucus layer [52]. In addition, its supplementation modulated the expression of genes related to intestinal inflammation and immune function, inhibited B-cell migration into the colon, and decreased common molecular proxies of inflammation.

## 4. Effects of *A. muciniphila* on Host Physiology

Human shotgun metagenomic studies have detected *A. muciniphila* in the majority of human stool samples [53]. While the biogeography of *A. muciniphila* within the human digestive tract is relatively uncharacterized, it is consistently detected in human colonic mucosal samples, and murine studies have found that *Akkermansia* is enriched in the colonic mucosa compared to the colonic lumen, consistent with its role in mucin degradation [54,55]. Interestingly, *Akkermansia* was also enriched in the small intestine compared to the colon, suggesting its potential to shape physiological processes in the small intestine [54]. Its distinctive traits as a mucin-degrading bacterium make it a crucial contributor to gut homeostasis and metabolism.

### 4.1. Current Methods Employed in the Study of A. muciniphila

To fully understand the physiologic role of *A. muciniphila*, researchers have utilized a combination of culture-based techniques, molecular methods, and experimental models. Anaerobic culture-based techniques have facilitated the isolation, culture, and comprehensive characterization of *A. muciniphila*, including its metabolic processes and reactions to environmental influences such as antibiotics and nutritional modifications. Initially, *A. muciniphila* was difficult to culture due to its classification as a strict anaerobe and its reliance on mucin as a principal nutrition source. After it was first discovered, specialized growth media enriched with mucin was found to enable the bacteria to flourish, resulting in the formation of visible colonies on solid media [56]. Microbial co-culture experiments, in which *A. muciniphila* is cultivated with other gut microbiota, allow for insights into the interaction of *A. muciniphila* with other gut microbes [57,58]. While culture-based techniques are important, they are limited in providing an accurate representation of the complexity of the gut microbiome [59]. The incorporation of molecular approaches is therefore important for a thorough understanding of the interactions of *A. muciniphila* and its role in the gut.

Molecular methods, including 16s rRNA gene sequencing, shotgun metagenomics, and metatranscriptomics, are widely used to characterize microbial composition and function of complex communities. 16s rRNA gene sequencing utilizes the sequences of the variable regions of the 16S rRNA gene to infer phylogeny, allowing for a comprehensive assessment of microbial diversity and the abundance of specific taxa, including *A. muciniphila*, in both human and animal microbiomes [60]. Shotgun metagenomic sequencing involves analyzing the entire DNA of a sample. While 16s rRNA sequencing focuses only on bacterial taxonomy, metagenomics also provides insights into potential functions. Genes involved in mucin degradation, host interaction, and other important metabolic processes of *A. muciniphila* have been discovered in this way [56,60,61]. For example, a metagenomic analysis of the gut microbiota in individuals with type 2 diabetes identified *A. muciniphila*-associated genes that may enhance insulin sensitivity [62,63,64]. Metatranscriptomics involves analyzing RNA transcripts from complex communities to provide an overview of active gene expression under various physiological conditions. For example, it has been used to analyze the gene expression profiles of *A. muciniphila* within the gut microbiota of mice fed high-fat diets. These investigations demonstrated that *A. muciniphila* modifies its metabolism in response to alterations in the host’s diet, suggesting its potential involvement in the metabolic outcomes of dietary changes [5].

Animal models are essential for investigating *A. muciniphila* since they allow for the characterization of host–microbe interactions in the setting of metabolic and gastrointestinal diseases. Mice and rats have been extensively utilized to examine the impacts of *A. muciniphila* on obesity, insulin resistance, immunity, gut physiology, and gut microbiota composition [4,65,66,67]. Gnotobiotic mice, reared in a sterile environment and lacking gut microbiota until colonization with defined microbes, have demonstrated significant utility in investigating the physiologic effects of *A. muciniphila* [68,69]. Co-culture techniques, in which *A. muciniphila* is cultivated with intestinal epithelial cells, permit an in-depth examination of its interactions with host cells to complement animal studies. Such in vitro studies have shown that *A. muciniphila* has a protective function in gut barrier integrity [11,70]. Moreover, organ-on-a-chip technologies have also been utilized to simulate gut function and characterize the behavior of *A. muciniphila* in various experimental settings [71].

### 4.2. Intestinal Barrier Integrity

The intestinal epithelial barrier is composed of tight junctions, adherens junctions, and desmosomes. A loss of barrier integrity is a feature of inflammatory bowel disease and other disorders, including metabolic syndrome [72]. While excessive mucin degradation can disrupt the host mucus layer’s protective effects and promote disease [61], *A. muciniphila* has been reported to promote intestinal barrier function [73]. Specific proteins in the outer membrane of *A. muciniphila*, including Amuc_1100, facilitate attachment to intestinal epithelial cells [74]. *A muciniphila* and Amuc_1100 have been shown to reduce circulating levels of lipopolysaccharides (LPS)—a marker of intestinal permeability that is associated with inflammation and metabolic diseases—in the setting of high-fat diet induced obesity or dyslipidemia due to apoprotein E deficiency [75,76]. One potential mechanism of these effects is the production of short-chain fatty acids (SCFAs), primarily acetate and propionate, following the degradation of host mucosal glycoproteins by *A. muciniphila* [22]. *A. muciniphila* may also be involved in cross-feeding interactions with butyrate-producing bacteria, such as *Faecalibacterium prausnitzii*, that promote the production of butyrate, another SCFA that is known to be a key modulator of intestinal health [77,78]. As the SCFA products of *A. muciniphila* mucin degradation are goblet cells’ preferred energy source, mucus synthesis by goblet cells is subsequently boosted. One study showed that the colonization of mice with *A. muciniphila* increased the number of colonic goblet cells and upregulated genes encoding mucin, including muc1, muc5, and muc13 [79]. *A. muciniphila* has been reported to protect against radiation-induced intestinal injury, with propionic acid derived from *A. muciniphila* found to enhance tight junction and mucin expression via G-protein-coupled receptor 43 (GPR43) [80]. *A. muciniphila* may also induce tight junction expression in intestinal epithelial cells via Toll-like receptor 2 (TLR2)-mediated pathways involving Amuc_1100 [66].

### 4.3. Immunomodulatory Properties

*A. muciniphila* colonization has been reported to stimulate adaptive immune responses even during homeostasis [81]. In contrast to many anti-commensal responses that involve the T-cell-independent production of IgA antibodies, *A. muciniphila* induces the production of IgG1 antibodies by plasma B cells in conjunction with follicular helper CD4+ T cells. While this was the only T-cell fate in gnotobiotic mice, mice with conventional gut microbiota also showed an expansion of pro-inflammatory T-cell populations reactive against *A. muciniphila*. In colitis models, *A. muciniphila* has been reported to induce the production of regulatory T-cell populations that attenuate intestinal inflammation [82]. The outer membrane of *A. muciniphila* contains LPS, but with structural differences compared to LPS derived from other pathogenic Gram-negative bacteria, which results in the comparatively weak activation of Toll-like receptor 4 (TLR4) [11]. However, *A. muciniphila* may modulate immune responses through the activation of TLR2 [66]. *A. muciniphila* also influences immune responses through the production of SCFAs that interact directly with intestinal epithelial and immune cells. These effects can be mediated by the interaction of acetate and propionate with G-protein-coupled receptors (Gprs) 41 and 43, which are located on diverse cell types, including enterocytes, colonocytes, neutrophils, enteroendocrine cells, and neurons [83]. SCFAs enhance intestinal stem cell proliferation in a Gpr41/43-dependent manner and promote intestinal epithelial regeneration [3]. Under certain conditions, there is evidence for pro-inflammatory actions of SCFAs [84]; however, for the most part, SCFAs and in particular butyrate have been found to confer an anti-inflammatory effect on the host, binding to Gpr109a on colonic macrophages and dendritic cells to induce the differentiation of Treg cells and IL-10-producing T cells. Furthermore, the butyrate interaction with Gpr109a gives rise to IL-18 production in colonocytes and mediates a butyrate-dependent protective effect in colitis models [85].

### 4.4. Lipid and Glucose Metabolism

Studies have shown that *A. muciniphila* can reduce fat accumulation in various models, particularly weight gain models induced by a high-fat diet (HFD) [86]. *A. muciniphila* is also associated with reduced triglyceride levels in models of hyperlipidemia and reduced expression of genes critical for the synthesis of triglycerides and lipids [87,88]. More studies are beginning to elucidate the specific mechanisms through which *A. muciniphila* influences lipid metabolism. During the fermentation of mucin, *A. muciniphila* produces SCFAs such as acetate and propionate, which modulate lipid metabolism by influencing the expression of genes related to fat storage and utilization [86,89]. Amuc_1100, a membrane protein from *A. muciniphila*, facilitates lipolysis and the browning of adipocytes via the activation of the AC3/PKA/HSL pathway, which is critical for the breakdown of stored fats [90]. Interestingly, extracellular vesicles derived from *A. muciniphila* were sufficient to improve lipid metabolism and reduce body weight gain in HFD-fed mice [91].

*A. muciniphila* has also been reported to modulate glucose metabolism and insulin sensitivity. In a study that utilized liquid chromatography and mass spectrometry, P9, a protein secreted by *A. muciniphila*, was found to induce thermogenesis and increase glucagon-like peptide-1 (GLP-1) secretion [92]. Moreover, SCFAs derived from mucin degradation regulated appetite by stimulating the release of GLP-1 and other gastrointestinal hormones by enteroendocrine cells [92]. Another study also provided evidence that *A. muciniphila* promotes insulin sensitivity and metabolic health through increased energy expenditure [93].

### 4.5. Effects on the Host Mediated by Interactions with Other Gut Microbes

*A. muciniphila* can influence host physiology through its effects on other microbes. In humans, a reduced *A. muciniphila* abundance is associated with dysbiosis across diverse gastrointestinal and non-gastrointestinal disorders [94]. *A. muciniphila* administration in many disease models has been correlated with improved dysbiosis. One study utilized a HFD-induced obesity mouse model to study the effects of *A. muciniphila* strains on obesity-related dysbiosis [88]. The EB-AMDK 27 strain decreased the abundance of the genus *Tyzzerella*, which is correlated with an increased risk of metabolic diseases, including obesity. Another more recent study examined *A. muciniphila*’s effects on metabolic-associated fatty liver disease (MAFLD) with three cohorts: low-fat diet-fed mice, HFD-fed mice, and HFD-fed mice gavaged orally with *A. muciniphila* (HA) [95]. They found that when compared to the HFD-fed mice, the HA group had a decreased *Tyzzerella* abundance and increased *Ruminiclostridium*, *Osclibacter*, *Allobaculum*, *Anaeroplasma*, and *Rikenella* abundances. *A. muciniphila* has also been found to improve dysbiosis in murine models of liver injury induced by alcohol and HFD/CCl4, including the enrichment of *Paramuribaculum intestinale* and *Bacteroides ovatus* as well as reductions in pathogenic bacteria within the Proteobacteria phylum [96,97].

*A. muciniphila*’s effects on other gut microbes have also been characterized in inflammatory disorders. A recent study examined a murine model of systemic lupus erythematosus [98]. While *A. muciniphila* did not significantly alter alpha diversity or microbial metabolic function, the treatment did result in a more complex network between members of the microbiome, which was suggested to contribute to decreased inflammation. *A. muciniphila* was also shown to significantly modulate the microbiota network in a study examining dextran sodium sulfate (DSS)-induced colitis, including the expansion of *Lactobacillus murinus* [99]. In addition, *A. muciniphila* altered the microbiome in mouse models of persistent inflammation, immunosuppression, and catabolism syndrome [100]. Both live and pasteurized *A. muciniphila* reduced the presence of bacteria associated with disease and inflammation and also increased the levels of *Muribaculaceae*, *Lachnospiraceae*, *Lachnoclostridium*, and *Parabacterbides goldsteinii*.

A recent study has also examined *A. muciniphila*’s ability to inhibit bacterial virulence genes, specifically in *Fusobacterium nucleatum*, a causative agent of periodontitis [101]. *A. muciniphila* inhibited the growth of *F. nucleatum* in co-culture and mouse models by downregulating the inflammatory TLR/MyD88/NF-κB pathway. They also found that *A. muciniphila* had a similar effect on other pathogenic microbes involved in the pathogenesis of periodontitis. The ability of *A. muciniphila* and its metabolites to inhibit other microbial genes is a promising sign of its ability to positively affect health outcomes related to microbe-mediated diseases. A summary of the effects of A. muciniphila can be found in Table 1.

## 5. *A. muciniphila* in Health and Disease

### 5.1. Human Studies

**Figure 1:**
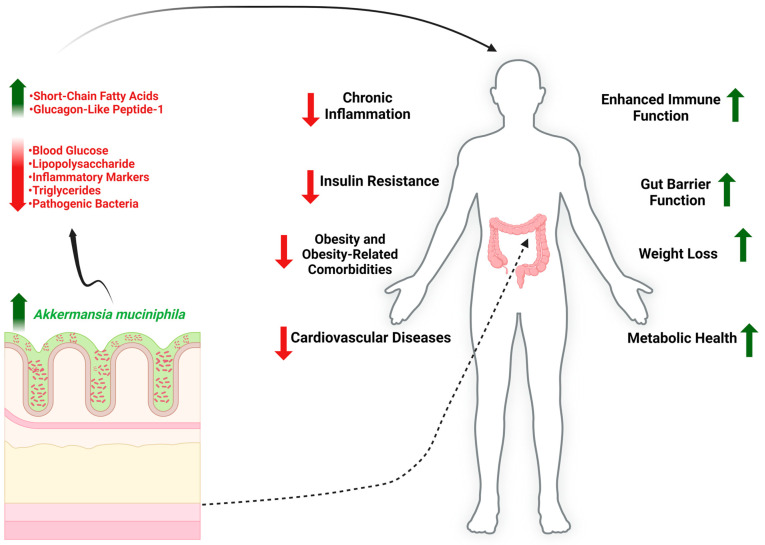
Beneficial effects of *Akkermansia muciniphila* reported on animal models and associations with improved health in human studies. Green up arrow indicates ‘increase/improvement’; red down arrow indicates ‘decrease/reduction’.

Numerous observational studies have noted the associations of *A. muciniphila* abundance with various factors, including but not limited to metabolic health [66,102], body weight [72], drug and antibiotic administration [41,66], and disease status [66,102,103,104,105,106] (Figure 1). In particular, the relative abundance of *A. muciniphila* has been found to be reduced in patients with type 1 [66] and type 2 diabetes [102], inflammatory bowel disease [66,103,106], and obesity [72], and conversely enriched in colorectal cancer patients [104] (Table 2). Moreover, the abundance of *A. muciniphila* has been negatively correlated with multiple disease-related factors, including pain in irritable bowel syndrome [107], clinical flares of ulcerative colitis [108], the duration of clinical remission of fasting blood glucose levels, and body weight [72]. An enriched *A. muciniphila* composition has been observed in the gut microbiome of patients deriving a clinical benefit from checkpoint inhibitor immunotherapy [109] and patients taking the glucose-lowering drug metformin [66]. Limited evidence also suggests that its abundance increases with bariatric surgery [110] and the administration of broad-spectrum antibiotics [41]. Additionally, an increased abundance of *A. muciniphila* has been observed in patients with mild cognitive impairment who received a modified Mediterranean–ketogenic diet that ameliorated markers of Alzheimer’s disease [111].

### 5.2. Obesity and Metabolic Disorders

Studies have repeatedly demonstrated reduced levels of *Akkermansia* in obese mice and humans and an inverse correlation between *A. muciniphila* abundance and metabolic health [2,4,63,112,113]. In animal models of diet-induced metabolic disorder, the administration of *A. muciniphila* reduced adiposity and hyperglycemia [4,66]. *A. muciniphila* treatment also attenuated fatty liver disease and lowered serum triglyceride levels [114]. Interestingly, metformin treatment of mice fed a high-fat diet increased *A. muciniphila* abundance, which has been proposed as a mechanism of the therapeutic effect of metformin [67]. The beneficial effect of *A. muciniphila* does not require live bacteria. Pasteurized *A. muciniphila* reduced the overall adiposity index in comparison to the control group (untreated HFD group) at the 14-day endpoint without affecting food intake [115]. The beneficial effect was attributed to the outer membrane protein Amuc_1100, which was heat stable and interacted with TLR2 to improve gut barrier function. Moreover, *Akkermansia* is enriched after gastric bypass surgery and has been proposed to mediate some of the beneficial effects of surgery on metabolic disease [4,112,116,117]. Colonization of germ-free mice with the microbiome of mice following gastric bypass surgery resulted in reduced weight gain and adiposity compared to recipients of the microbiome from sham-operated mice, which were associated with increased *Akkermansia* [116]. Colonization of mice with the post-gastric surgery microbiota from MAFLD patients resulted in increased *Akkermansia* abundance, reduced susceptibility to HFD-induced weight gain and steatohepatitis, and reduced glucose-dependent insulinotropic polypeptide (GIP) levels [117]. These findings collectively have established a strong relationship between *A. muciniphila* and metabolic health.

### 5.3. Cardiovascular Diseases

*A. muciniphila* has also been associated with protection against cardiovascular disease. The administration of *A. muciniphila* ameliorated the development of atherosclerosis in mice with apolipoprotein E deficiency [76]. This was accompanied by a lower macrophage quantity and decreased levels of inflammatory markers such as ICAM-1, TNF-α, and MCP-1. This effect on animal models has been associated with reduced circulating levels of trimethylamine *N*-oxide, a microbiome-derived molecule associated with atherosclerosis [118]. Additionally, there was a negative correlation between *A. muciniphila* and the progression of abdominal aortic aneurysm (AAA) in a murine model [65]. The administration of *A. muciniphila* inhibited development of AAA and was associated with improvements in circulating inflammatory markers.

### 5.4. Inflammatory Disorders

*A. muciniphila* has been postulated to have a protective role in inflammatory bowel disease. In support of this, the administration of *A. muciniphila* has been reported to ameliorate colitis in two mouse models: dextran sodium sulfate (DSS)-induced colitis and T-cell-transfer-induced colitis [82]. A magnetized *A. muciniphila* using novel nanoparticles (AKK@MFe_3_O_4_), allowing magnet-guided colonization of the gastrointestinal tract, similarly showed a beneficial effect on DSS-induced colitis [99]. There has also been interest in inflammatory disorders outside the intestines. One study of acetaminophen-induced liver injury noted that *A. muciniphila* treatment decreased inflammation and the activation of inflammatory cells, resulting in a significant improvement in comparison to non-treated mice [89]. In another study examining sepsis-induced acute lung injury models in mice, oral administration of Amuc_1100, an outer membrane protein of *A. muciniphila*, was found to balance dysbiosis, decrease inflammatory cytokine levels, and improve health outcomes [119]. In an additional study, the administration of *A. muciniphila* strain EB-AMDK19 improved the Th1/Th2 balance in mouse models, improving atopic dermatitis symptoms [120].

### 5.5. Malignancy

The administration of *A. muciniphila* has also shown potential in modulating cancer development and treating toxicity. One study found that colitis-associated colorectal cancer was mitigated by the administration of *A. muciniphila* and Amuc_1100, which was associated with the induction of cytotoxic T lymphocytes [121]. Another study examined the tumor microbiome of lung cancer mouse models and found that tumorigenesis was inhibited after the administration of *A. muciniphila* [122]. *A. muciniphila* upregulated several metabolic pathways, including glycolysis, fatty acid biosynthesis, and glutamine that had previously been downregulated in the mouse lung cancer models. *A. muciniphila* strain BAA-835 has been studied in a mouse model of mucositis, an inflammation of the mouth and GI tract caused by cancer treatment. This strain attenuated chemotherapy-induced intestinal injury and downregulated the production of inflammatory cytokines, including IL1β, IL6, and TNFα [123].

### 5.6. Clostridioides Difficile Infection

*A. muciniphila* has been reported to be enriched in *Clostridioides difficile*-infected (CDI) patients [124,125]. These two studies were conducted by the same laboratory and featured distinct patient populations—elderly CDI patients (>65) and patients ranging from 18 to 60 years—at two separate time points. These findings raise questions about the relationship of *A. muciniphila* with CDI pathogenesis. A recent study modeled CDI in C57BL/6 mice and found that *A. muciniphila* administration improved CDI-associated dysbiosis [126]. In particular, the *Blautia* and *Parabacteroides* genera were enriched in the *A. muciniphila*-treated group, while there was a decrease in the abundance of microbes associated with CDI, including members of the *Enterobacteriaceae* and *Enterococcaceae* families.

**Table 2:** *A. muciniphila* in health and disease. Symbol guide: +: happening concurrently. a -> b: ‘b’ happens after ‘a’.

Section	Experimental Conditions	Main Beneficial Effects
Obesity and Metabolic Disorders	- C57BL/6 mice: HFD -> 4 weeks of daily oral gavage of *A. muciniphila* (live or heat-killed) [4] - C57BL/6J mice: HFD -> 4 weeks of daily oral gavage of *A. muciniphila* (live or pasteurized) [66] - Fatty liver disease model: C57BL/6N mice received 10 weeks of daily oral gavage of *A. muciniphila* + HFD [114] - Roux-en-Y gastric bypass (RYGB) model: colonization of germ-free mice with the microbiome of mice following gastric bypass surgery [116] - Post-bariatric surgery treatment model for nonalcoholic fatty liver disease: colonization of C57/Bl6 mice with the post-gastric surgery microbiota from NAFLD patients [117]	- Reduces adiposity and hyperglycemia [4,66] - Attenuates fatty liver disease and lowers serum triglyceride levels [114] - Reduces weight gain and adiposity, associated with increased *A. muciniphila* abundance [116] - Increased *A. muciniphila* abundance reduces the susceptibility to HFD-induced weight gain and steatohepatitis, and reduces GIP levels [117]
Cardiovascular Diseases	- Abdominal aortic aneurysm model: C57BL/6J mice with induced abdominal aortic aneurysm (AAA) + one-time oral gavage *A. muciniphila* [65] - Apolipoprotein E-deficient mice (dyslipidemia model): 8 weeks of daily oral gavage of *A. muciniphila* [76]	- Inhibits the development of AAA and is associated with improvements in circulating inflammatory markers [65] - Ameliorates the development of atherosclerosis in mice with apolipoprotein E deficiency, lowers the macrophage quantity, and decreases the levels of inflammatory markers such as ICAM-1, TNF-α, and MCP-1 [76]
Inflammatory Disorders	- WT and TLR4−/− mice with *A. muciniphila* + DSS-induced colitis [82] - Acute liver injury mouse model (24 h after 300 mg/kg APAP dosing) + *A. muciniphila* [89] - Cecal ligation and puncture (CLP)-induced sepsis model: oral gavage of Amuc_1100 in mice before the procedure to induce sepsis [119] - Mouse atopic dermatitis (AD) model: NC/Nga mice with induced AD -> 6 weeks of daily oral gavage of *A. muciniphila* [120]	- Ameliorates colitis in two mouse models, including dextran sodium sulfate (DSS)-induced colitis and T-cell-transfer-induced colitis [82] - Decreases inflammation and the activation of inflammatory cells [89] - The administration of Amuc_1100 (an outer membrane protein of *A. muciniphila*) balances dysbiosis, decreases inflammatory cytokine levels, and improves health outcomes of sepsis-induced acute lung injury [119] - The administration of *A. muciniphila* strain EB-AMDK19 improves the Th1/Th2 balance in mouse models, improving atopic dermatitis symptoms [120]
Malignancy	- DSS-induced colitis model in C57BL/6J mice: one-time oral gavage (*A. muciniphila* or Amuc_1100) -> DSS administration [121] - Tumor microbiome of a mouse lung cancer model: C57BL/6 mice received oral gavage of *A. muciniphila* every 2 days -> induced tumor model [122] - Mouse mucositis model (an inflammation of the mouth and GI tract caused by cancer treatment): Balb/c mice were gavaged with *A. muciniphila* strain BAA-835 [123]	- Its administration mitigates colitis-associated colorectal cancer, as *A. muciniphila* is associated with the induction of cytotoxic T lymphocytes [121] - Inhibits tumorigenesis by upregulating several metabolic pathways, including glycolysis, fatty acid biosynthesis, and glutamine metabolism, that had previously been downregulated in the mouse lung cancer models [122] - Attenuates chemotherapy-induced intestinal injury and downregulates the production of inflammatory cytokines, including IL1β, IL6, and TNF [123]
*Clostridioides difficile* Infection	CDI infection model: C57BL/6 mice received daily oral gavage of *A. muciniphila* for 2 weeks [126]	Improves CDI-associated dysbiosis [126]
Age-Related Changes	Aging mouse model: Ercc1−/Δ7 mice received oral gavage of *A. muciniphila* 3 times a week for 10 weeks [52]	Induces thickening of the colonic mucus layer, modulates the expression of genes related to intestinal inflammation and immune function, inhibits B-cell migration into the colon, and decreases common molecular proxies of inflammation [52]

## 6. Therapeutic Applications of *A. muciniphila*

Many studies have been conducted to evaluate the therapeutic potential of *A. muciniphila* in a variety of disease contexts based on the existing literature that supports its beneficial health effects (Table 3).

### 6.1. Interventions to Modulate A. muciniphila Abundance

Ongoing research has identified several strategies to increase the abundance of *A. muciniphila* in the gut, including dietary adjustments, pharmaceuticals, prebiotics, and fecal microbiota transplantation. Diet greatly impacts the gut microbiota, and dietary supplements could significantly affect the abundance of *A. muciniphila*. A study by Schneeberger et al. (2015) revealed that a meal supplemented with fish oil significantly elevated *A. muciniphila* levels in mice, but a lard-laden diet caused a reduction [63]. This indicates that the type of dietary fats ingested can influence the amount of *A. muciniphila*, potentially enhancing gut barrier function and diminishing inflammation. Shang et al. indicated that dietary polysaccharides from *Enteromorpha clathrata* (seaweed) enhance the proliferation of *A. muciniphila* and other advantageous gut bacteria, such as *Bifidobacterium* and *Lactobacillus* [127]. Metformin, a pharmaceutical agent used in the management of type 2 diabetes, has also been linked to an increase in *A. muciniphila* abundance [19,67]. Fecal microbiota transplantation (FMT) has the potential to restore a healthy gut microbiome, including the re-establishment of beneficial bacteria like *A. muciniphila*. FMT was found to positively influence the abundance of *A. muciniphila* in patients with type 2 diabetes mellitus [128,129]. In mice, FMT from young mice to old mice resulted in improvements in several age-related parameters, including the loss of intestinal barrier structure, inflammation, and glucose insensitivity, which were associated with higher *A. muciniphila* levels [130].

**Table 3:** Therapeutic applications of *A. muciniphila*. Symbol guide: a -> b: ‘b’ happens after ‘a’.

Section	Experimental Conditions	Main Beneficial Effects
Products of *A. muciniphila*	- Tumorigenesis models in mice: administration of purified Amuc_2172 [131] - Sepsis models: specific pathogen-free C57BL/6 mice: Induced sepsis model -> 7 days of oral gavage of Arg-Lys-His [132] - Gut permeability model in C57BL/6 mice: HFD -> a 2-week administration of *A. muciniphila* extracellular vesicles [133]	- Amuc_2172 (acetyltransferase from *A. muciniphila*) inhibits tumorigenesis in colorectal cancer models by upregulating the production of heat-shock protein 70 (HSP70) and improving the immune response through increased CD8+ cytotoxic T lymphocyte activity [131] - The administration of Arg-Lys-His (Novel tripeptide derived from *A. muciniphila*) reduces inflammation symptoms through TLR4 inhibition [132] - The administration of *A. muciniphila* extracellular vesicles promotes gut barrier integrity [133]
Probiotic Potential	- Type 2 diabetes clinical trial: 12-weeks of administration of a probiotic formulation that included *A. muciniphila* [134] - Muscle function clinical trial: 12-week administration of pasteurized *A. muciniphila* HB05 (HB05P) [135] - Respiratory symptom clinical trial: 4 to 12 weeks of administration of ETB-F01, a formulation containing heat-killed *A. muciniphila* strain EB-AMDK19 [136]	- A significant reduction in postprandial glucose levels, improves insulin sensitivity and other metabolic parameters [134] - Significant improvements in muscle strength, muscle function, and physical performance metrics [135] - Significant improvements in the Breathlessness, Cough, and Sputum Scale score, indicating a reduction in respiratory symptoms and enhancement of lung function [136]

### 6.2. Products of A. muciniphila

The therapeutic potential of *A. muciniphila*’s metabolites has also been of interest. Multiple animal model studies have demonstrated that Amuc_1100 can improve adiposity, insulin resistance, and dyslipidemia, as well as reduce colitis-associated malignancy and sepsis-induced lung injury [66,75,90,115,119,121]. A newly discovered acetyltransferase from *A. muciniphila*, Amuc_2172, was found to inhibit tumorigenesis in colorectal cancer models [131]. Amuc_2172 specifically upregulated the production of heat-shock protein 70 (HSP70), which in turn improved the immune response through increased CD8+ cytotoxic T lymphocyte activity. In a sepsis study, murine and pig models were treated with the novel tripeptide Arg-Lys-His derived from *A. muciniphila*, which was reported to reduce inflammation symptoms through TLR4 inhibition [132]. Furthermore, it has been demonstrated that the administration of extracellular vesicles formed from *A. muciniphila* influences gut permeability and promotes gut barrier integrity, suggesting that these vesicles could be used as a therapeutic approach [133]. These results support the potential development of novel therapies based upon *A. muciniphila*’s metabolites and membrane vesicles, though further research is needed to translate these findings into clinical applications.

### 6.3. Probiotic Potential

There is now extensive literature demonstrating the beneficial effects of *A. muciniphila* administration in mouse models of diseases characterized by dysbiosis, inflammation, and a dysregulated immune response (see Section 3 and Section 4). While there exists an abundance of observational and correlational human studies pointing to *A. muciniphila*’s potential role in human health, there have been far fewer human trials that directly evaluate its health benefits. One significant challenge lies in the dependence on animal-derived compounds in the growth medium for *A. muciniphila*; however, recent advancements have led to the development of a synthetic medium that produces bacteria that is safe when administered to human subjects. *A. muciniphila*’s high oxygen sensitivity is yet another barrier to its use in live human subjects [66]. Overcoming these barriers, an initial clinical trial tested the use of an *A. muciniphila* supplement in a cohort of overweight and obese insulin-resistant human participants [103]. They confirmed the safety of oral supplementation of both live and pasteurized *A. muciniphila*. Daily oral supplementation of 10^10^ live or pasteurized *A. muciniphila* for 3 months reduced insulinemia, plasma cholesterol levels, body weight, and the levels of relevant blood markers of liver dysfunction and inflammation compared to the placebo. The overall gut microbiome structure was unchanged between groups. Since this initial study, at least three additional clinical trials of *A. muciniphila* have been published [134,135,136]. An investigation into the effects of a probiotic formulation containing *A. muciniphila* on postprandial glucose levels in individuals with type 2 diabetes revealed significant reductions in postprandial glucose levels in these patients [134]. Additionally, improvements were observed in insulin sensitivity and other metabolic parameters, suggesting that the probiotic formulation may enhance the overall metabolic health of individuals with diabetes. Kang et al. examined the effects of pasteurized *A. muciniphila* HB05 (HB05P) on muscle strength and function in elderly patients in the absence of exercise over 12 weeks. The results indicated significant improvements in muscle strength, muscle function, and physical performance metrics among participants receiving HB05P compared to those in the placebo group [135]. An additional clinical study investigated the effects of ETB-F01, a formulation containing heat-killed *A. muciniphila* strain EB-AMDK19, on patients experiencing respiratory symptoms. Participants that received daily oral administration of ETB-F01 showed significant improvements in the Breathlessness, Cough, and Sputum Scale score, indicating improvements in respiratory symptoms and enhancement of lung function [136]. The findings of these trials could have significant implications for the management of diabetes and other health conditions, supporting the incorporation of probiotics like *A. muciniphila* into treatment regimens to enhance patient outcomes.

## 7. Limitations of Current Studies on *A. muciniphila*

Although *A. muciniphila* exhibits significant potential as a probiotic, there are various limitations in understanding its biology, ecology, and therapeutic applications. These limitations are a result of methodological difficulties, diversity in study designs, and the complexities of the gut microbiome. It has proven difficult to culture *A. muciniphila* under in vitro conditions that accurately mimic its natural gut habitat. As an anaerobic bacterium that lives in the mucus layer of the intestinal epithelium, it is often difficult to replicate that habitat outside of the host [114]. Also, variations among the clinical isolates may affect their growth characteristics and interactions with other gut microbes, which further complicate the interpretation of the experimental results [13]. Much of the existing literature on *A. muciniphila* consists of mouse studies [137]. Although these studies are important and offer significant insights, they may not completely apply to humans due to differences in physiology, gut microbiota composition, and diet. Existing human studies have been characterized by limited sample sizes and varying methodologies [65,95]. Heterogeneity in diet and gut microbiome could make it difficult to come to general conclusions about the effects of *A. muciniphila* across different human populations [36]. The interactions of *A. muciniphila* with other gut microbiota add another layer of complexity. Research has indicated that the presence of other bacterial species can inhibit the growth of *A. muciniphila* in co-culture systems, suggesting competitive dynamics that may influence its abundance and behavior in the gut [138]. Optimized methodologies, standardized study designs, and interventional translational studies will be crucial for advancing our understanding of the roles of *A. muciniphila* in health and disease.

## 8. Future Directions

*A. muciniphila* plays a crucial role in maintaining gut barrier integrity and modulating immune responses as a result of its capacity to degrade mucin and produce beneficial metabolites such as SCFAs [2,114]. The increasing amount of evidence, demonstrating *A. muciniphila*’s health advantages, makes it a viable option for treating various metabolic diseases. Understanding the distinct strains of *A. muciniphila*, their functional diversity, and their interactions with other gut microbes should be the main goals of future research [27,139]. Additionally, large-scale clinical trials are needed to confirm the efficacy of *A. muciniphila* supplementation in diverse populations and its long-term effects on metabolic health [8,128]. Furthermore, the interactions between *A. muciniphila*, the host immune system, and other gut microbes should be explored to provide additional insights into its therapeutic potential [57,74]. Understanding the impact of diet on *A. muciniphila* abundance and functionality could potentially result in customized dietary recommendations that would promote gut health [10,113]. Lastly, investigating the synergistic benefits of *A. muciniphila* in combination with other microbiome-based therapeutics, including prebiotics and probiotics, may unveil novel strategies for addressing complex metabolic disorders.

## Figures and Tables

**Table 1:** Effects of *A. muciniphila* on host physiology. Symbol guide: +: happening concurrently. a -> b: ‘b’ happens after ‘a’.

Section	Experimental Conditions	Main Beneficial Effects
Intestinal Barrier Integrity	- Mouse nonalcoholic fatty liver disease model: *A. muciniphila* and Amuc_1100 + a 10-week HFD [75] - Mouse atherosclerosis model: 8 weeks of daily oral gavage of *A. muciniphila* + dyslipidemia (due to apoprotein E deficiency) [76]	Reduced circulating levels of LPS (a marker of intestinal permeability that is associated with inflammation and metabolic diseases) [75,76]
	Irradiation-induced injury model: BALB/c mice received daily gavage of *A. muciniphila* for 28 days -> induced irradiation injury [79]	Increased number of colonic goblet cells and upregulated expression of the genes encoding mucin, including muc1, muc5, and muc13 [79]
	- Mouse model of obesity and diabetes model: HFD -> 5 weeks of daily oral gavage of pasteurized (30 min at 70 °C) *A. muciniphila* and Amuc_1100 [66] - Mouse irradiation-induced injury model: 1 × 10^8^ CFUs of *A. muciniphila* by gavage for 28 consecutive days before and 3 days after abdominal interventional radiology (IR) at 8 Gy [80]	- Potentially induces tight junction protein expression in intestinal epithelial cells [66] - Protects against radiation-induced intestinal injury [80]
Immunomodulatory Properties	Mouse chronic intestinal inflammation model: WT and TLR4−/− mice with *A. muciniphila* + DSS-induced colitis [82]	Induces the production of regulatory T-cell populations that attenuate intestinal inflammation [82]
Lipid and Glucose Metabolism	- Obesity model in *Caenorhabditis elegans*: dilutions of *A. muciniphila* cell-free supernatant + a high-glucose diet [86] - Genetic-induced hyperlipidemia model in C57BL/6J mice: 12-week-old mice with a 2-week oral gavage of *A. muciniphila* + olive oil-induced hyperlipidemia [87] - Mouse obesity model: HFD -> 12 weeks, every 6 days, oral gavage of *A. muciniphila* [88] - Mouse acute liver injury model: (24 h after 300 mg/kg APAP dosing) + *A. muciniphila* [89] - Obesity model in C57BL/6 Mice: HFD + *A. muciniphila* or HFD + Amuc_1100 [90] - Administered extracellular vesicles derived from *A. muciniphila* [91]	- Reduces weight gain induced by an HFD [86] - Associated with reduced triglyceride levels in hyperlipidemia models [87] - Reduced expression of genes critical for triglyceride and lipid synthesis [88] - Produces SCFAs such as acetate and propionate that modulate lipid metabolism by influencing the expression of genes related to fat storage and utilization [89] - Amuc_1100 (a membrane protein from *A. muciniphila*) facilitates lipolysis and browning of adipocytes via the activation of the AC3/PKA/HSL pathway, which is critical for the breakdown of stored fats [90] - Improves lipid metabolism and reduces body weight gain in HFD-fed mice [91]
	- High-fat diet model in C57BL/6J mice: 14 weeks of daily oral gavage of *A. muciniphila* + HFD [92] - Obesity model in C57BL/6J mice: 5 weeks of daily oral gavage of *A. muciniphila* + HFD [93]	- P9 (*A. muciniphila* secreted protein) improves glucose homeostasis by inducing thermogenesis and increasing glucagon-like peptide-1 (GLP-1) secretion [92] - Regulates appetite through SCFAs produced from mucin degradation (stimulates enteroendocrine cells to release GLP-1 and other gastrointestinal hormones) [92] - Promotes insulin sensitivity and metabolic health through increased energy expenditure [93]
Interaction with Other Gut Microbes	- Mouse obesity model: HFD-induced obesity -> 12 weeks with 6 oral gavages each week of EB-AMDK 27 (specific *A. muciniphila* strain) [88] - Metabolic-associated fatty liver disease (MAFLD) model: specific pathogen-free C57BL/6 mice received daily oral gavage *A. muciniphila* + HFD for 21 weeks [95] - Alcoholic liver disease model in C57BL/6 nice: 15 days of daily oral gavage of *A. muciniphila* + NIAAA model (Induced alcohol liber injury) [96] - Liver fibrosis model in C57BL/6 mice: 4 weeks of daily oral gavage of *A. muciniphila* extracellular vesicles + HFD + carbon tetrachloride (induced liver fibrosis) [97]	- Decreases the prevalence of *Tyzzerella* (a genus correlated with an increased risk of metabolic diseases) [88] - Decreases the abundance of *Tyzzerella* and increases the abundance of *Ruminiclostridium*, *Osclibacter*, *Allobaculum*, *Anaeroplasma*, and *Rikenella* (genera associated with beneficial health effects) [95] - Improves dysbiosis, including an enrichment of *Paramuribaculum intestinale* and *Bacteroides ovatus* and a reduction in pathogenic bacteria within the *Proteobacteria* phylum [96,97]
	- Systemic lupus erythematosus model in MRL/lpr mice: oral gavage of *A. muciniphila* every 2 days for 7 weeks [98] - Dextran sodium sulfate (DSS)-induced colitis model in C57BL/6J mice: 8 days of DSS administration -> 2 days of oral gavage of *A. muciniphila* [99] - Persistent inflammation, immunosuppression, and catabolism syndrome (PICS) model in C57BL/6 mice: PICS model -> 2 oral gavages (live or pasteurized *A. muciniphila*) for 7 days [100]	- Administration resulted in a more complex network between members of the microbiome that may contribute to decreased inflammation [98] - Expands the population of the probiotic *Lactobacillus murinus* [99] - Reduces the presence of bacteria associated with disease and inflammation and increases the levels of *Muribaculaceae*, *Lachnospiraceae*, *Lachnoclostridium*, and *Parabacterbides goldsteinii* (genera associated with beneficial health effects) [100]
	Co-culture: 1 × 10^8^ CFUs/mL of *F. nucleatum* and 1 × 10^7^ CFUs/mL of *A. muciniphila* for 1–3 days; Specific pathogen-free BALB/c mice: antibiotics added to the water -> *A. muciniphila* + *F. nucleatum* applied 4 times a week for 8 weeks [101]	Inhibited the growth of *F. nucleatum* in co-culture and mouse models by downregulating the inflammatory TLR/MyD88/NF-κB pathway, with similar effects on other pathogenic microbes involved in periodontitis [101]

## Data Availability

No new data were created or analyzed in this study.

## Abbreviations

The following abbreviations are used in this manuscript:

AAA	Abdominal Aortic Aneurysm
AC3	Adenylate Cyclase
CCl4	Carbon Tetrachloride
CD4	Cluster of Differentiation 4
CD8	Cluster of Differentiation 8
CDI	*Clostridioides Difficile* Infection
CRISPR	Clustered Regularly Interspaced Short Palindromic Repeats
DSS	Dextran Sulfate Sodium
FMT	Fecal Microbiota Transplantation
GalNAc	*N*-Acetyl Galactosamine
GI	Gastrointestinal
GIP	Gastric Inhibitory Polypeptide
GlcNAc	*N*-Acetylglucosamine
GLP-1	Glucagon-Like Peptide-1
Gpr	G-Protein-Coupled Receptors
HA	HFD-Fed Mice Gavaged Orally with *A. Muciniphila*
HFD	High-Fat Diet
HSL	Hormone-Sensitive Lipase
HSP70	Heat Shock Protein 70
ICAM-1	Intercellular Adhesion Molecule 1
IgA	Immunoglobulin A
IgG1	Immunoglobulin G1
IL-10	Interleukin 10
IL-18	Interleukin-18
IL1β	Interleukin-1 Beta
IL6	Interleukin-6
LD	Linear Dichroism
LPS	Lipopolysaccharide
MAFLD	Metabolic-Associated Fatty Liver Disease
MCP	Monocyte Chemoattractant Protein
MyD88	Myeloid Differentiation Primary Response 88
NCBI	National Center for Biotechnology Information
NF-κB	Nuclear Factor Kappa B
PKA	Protein Kinase A
SCFAs	Short-Chain Fatty Acids
TLR2	Toll-Like Receptor 2
TLR4	Toll-Like Receptor 4
TNF-α	Tumor Necrosis Factor Alpha

## References

[1] Flint H.J., Scott K.P., Louis P., Duncan S.H. (2012). The role of the gut microbiota in nutrition and health. Nat. Rev. Gastroenterol. Hepatol..

[2] Naito Y., Uchiyama K., Takagi T. (2018). A next-generation beneficial microbe: *Akkermansia muciniphila*. J. Clin. Biochem. Nutr..

[3] Cani P.D., Bibiloni R., Knauf C., Waget A., Neyrinck A.M., Delzenne N.M., Burcelin R. (2008). Changes in gut microbiota control metabolic endotoxemia-induced inflammation in high-fat diet-induced obesity and diabetes in mice. Diabetes.

[4] Everard A., Belzer C., Geurts L., Ouwerkerk J.P., Druart C., Bindels L.B., Guiot Y., Derrien M., Muccioli G.G., Delzenne N.M. (2013). Cross-talk between *Akkermansia muciniphila* and intestinal epithelium controls diet-induced obesity. Proc. Natl. Acad. Sci. USA.

[5] Kumar R., Kane H., Wang Q., Hibberd A., Jensen H.M., Kim H.S., Bak S.Y., Auzanneau I., Bry S., Christensen N. (2022). Identification and Characterization of a Novel Species of Genus *Akkermansia* with Metabolic Health Effects in a Diet-Induced Obesity Mouse Model. Cells.

[6] Mueller K.D., Panzetta M.E., Davey L., McCann J.R., Rawls J.F., Flores G.E., Valdivia R.H. (2024). Pangenomic analysis identifies correlations between *Akkermansia* species and subspecies and human health outcomes. Microbiome Res. Rep..

[7] Derrien M., Belzer C., de Vos W.M. (2017). *Akkermansia muciniphila* and its role in regulating host functions. Microb. Pathog..

[8] Si J., Kang H., You H.J., Ko G. (2022). Revisiting the role of *Akkermansia muciniphila* as a therapeutic bacterium. Gut Microbes.

[9] Zhu L., Lu X., Liu L., Voglmeir J., Zhong X., Yu Q. (2020). *Akkermansia muciniphila* protects intestinal mucosa from damage caused by S. pullorum by initiating proliferation of intestinal epithelium. Vet. Res..

[10] van der Ark K.C.H., Aalvink S., Suarez-Diez M., Schaap P.J., de Vos W.M., Belzer C. (2018). Model-driven design of a minimal medium for *Akkermansia muciniphila* confirms mucus adaptation. Microb. Biotechnol..

[11] Reunanen J., Kainulainen V., Huuskonen L., Ottman N., Belzer C., Huhtinen H., de Vos W.M., Satokari R. (2015). *Akkermansia muciniphila* Adheres to Enterocytes and Strengthens the Integrity of the Epithelial Cell Layer. Appl. Environ. Microbiol..

[12] van Passel M.W., Kant R., Zoetendal E.G., Plugge C.M., Derrien M., Malfatti S.A., Chain P.S., Woyke T., Palva A., de Vos W.M. (2011). The genome of *Akkermansia muciniphila*, a dedicated intestinal mucin degrader, and its use in exploring intestinal metagenomes. PLoS ONE.

[13] Becken B., Davey L., Middleton D.R., Mueller K.D., Sharma A., Holmes Z.C., Dallow E., Remick B., Barton G.M., David L.A. (2021). Genotypic and Phenotypic Diversity among Human Isolates of *Akkermansia muciniphila*. mBio.

[14] Guo X., Li S., Zhang J., Wu F., Li X., Wu D., Zhang M., Ou Z., Jie Z., Yan Q. (2017). Genome sequencing of 39 *Akkermansia muciniphila* isolates reveals its population structure, genomic and functional diverisity, and global distribution in mammalian gut microbiotas. BMC Genom..

[15] Xing J., Li X., Sun Y., Zhao J., Miao S., Xiong Q., Zhang Y., Zhang G. (2019). Comparative genomic and functional analysis of *Akkermansia muciniphila* and closely related species. Genes Genom..

[16] Wu Z., Xiao Y., Zhou F., Chen J., Chen X., Hou A., Wang Y., Li Z. (2022). Pasteurized *Akkermansia muciniphila* Reduces Fat Accumulation via nhr-49-Mediated Nuclear Hormone Signaling Pathway in Caenorhabditis elegans. Molecules.

[17] Zhai R., Xue X., Zhang L., Yang X., Zhao L., Zhang C. (2019). Strain-Specific Anti-inflammatory Properties of Two *Akkermansia muciniphila* Strains on Chronic Colitis in Mice. Front. Cell. Infect. Microbiol..

[18] Ndongo S., Armstrong N., Raoult D., Fournier P.E. (2022). Reclassification of eight *Akkermansia muciniphila* strains and description of *Akkermansia* massiliensis sp. nov. and Candidatus *Akkermansia* timonensis, isolated from human feces. Sci. Rep..

[19] de la Cuesta-Zuluaga J., Mueller N.T., Corrales-Agudelo V., Velasquez-Mejia E.P., Carmona J.A., Abad J.M., Escobar J.S. (2017). Metformin Is Associated With Higher Relative Abundance of Mucin-Degrading *Akkermansia muciniphila* and Several Short-Chain Fatty Acid-Producing Microbiota in the Gut. Diabetes Care.

[20] Ouwerkerk J.P., van der Ark K.C.H., Davids M., Claassens N.J., Finestra T.R., de Vos W.M., Belzer C. (2016). Adaptation of *Akkermansia muciniphila* to the Oxic-Anoxic Interface of the Mucus Layer. Appl. Environ. Microbiol..

[21] Zhang T., Li Q., Cheng L., Buch H., Zhang F. (2019). *Akkermansia muciniphila* is a promising probiotic. Microb. Biotechnol..

[22] Ottman N., Davids M., Suarez-Diez M., Boeren S., Schaap P.J., Martins Dos Santos V.A.P., Smidt H., Belzer C., de Vos W.M. (2017). Genome-Scale Model and Omics Analysis of Metabolic Capacities of *Akkermansia muciniphila* Reveal a Preferential Mucin-Degrading Lifestyle. Appl. Environ. Microbiol..

[23] Kostopoulos I., Elzinga J., Ottman N., Klievink J.T., Blijenberg B., Aalvink S., Boeren S., Mank M., Knol J., de Vos W.M. (2020). *Akkermansia muciniphila* uses human milk oligosaccharides to thrive in the early life conditions in vitro. Sci. Rep..

[24] Louis P., Flint H.J. (2017). Formation of propionate and butyrate by the human colonic microbiota. Environ. Microbiol..

[25] Cani P.D., Depommier C., Derrien M., Everard A., de Vos W.M. (2022). *Akkermansia muciniphila*: Paradigm for next-generation beneficial microorganisms. Nat. Rev. Gastroenterol. Hepatol..

[26] Belzer C., Chia L.W., Aalvink S., Chamlagain B., Piironen V., Knol J., de Vos W.M. (2017). Microbial Metabolic Networks at the Mucus Layer Lead to Diet-Independent Butyrate and Vitamin B(12) Production by Intestinal Symbionts. mBio.

[27] Kirmiz N., Galindo K., Cross K.L., Luna E., Rhoades N., Podar M., Flores G.E. (2020). Comparative Genomics Guides Elucidation of Vitamin B(12) Biosynthesis in Novel Human-Associated *Akkermansia* Strains. Appl. Environ. Microbiol..

[28] Schrager J. (1970). The chemical composition and function of gastrointestinal mucus. Gut.

[29] Ribo S., Sanchez-Infantes D., Martinez-Guino L., Garcia-Mantrana I., Ramon-Krauel M., Tondo M., Arning E., Nofrarias M., Osorio-Conles O., Fernandez-Perez A. (2021). Increasing breast milk betaine modulates *Akkermansia* abundance in mammalian neonates and improves long-term metabolic health. Sci. Transl. Med..

[30] Yang Y., Zhong Z., Wang B., Xia X., Yao W., Huang L., Wang Y., Ding W. (2019). Early-life high-fat diet-induced obesity programs hippocampal development and cognitive functions via regulation of gut commensal *Akkermansia muciniphila*. Neuropsychopharmacology.

[31] Perez-Monter C., Alvarez-Arce A., Nuno-Lambarri N., Escalona-Nandez I., Juarez-Hernandez E., Chavez-Tapia N.C., Uribe M., Barbero-Becerra V.J. (2022). Inulin Improves Diet-Induced Hepatic Steatosis and Increases Intestinal *Akkermansia* Genus Level. Int. J. Mol. Sci..

[32] Pontifex M.G., Mushtaq A., Le Gall G., Rodriguez-Ramiro I., Blokker B.A., Hoogteijling M.E.M., Ricci M., Pellizzon M., Vauzour D., Muller M. (2021). Differential Influence of Soluble Dietary Fibres on Intestinal and Hepatic Carbohydrate Response. Nutrients.

[33] Roshanravan N., Mahdavi R., Alizadeh E., Ghavami A., Rahbar Saadat Y., Mesri Alamdari N., Alipour S., Dastouri M.R., Ostadrahimi A. (2017). The effects of sodium butyrate and inulin supplementation on angiotensin signaling pathway via promotion of *Akkermansia muciniphila* abundance in type 2 diabetes; A randomized, double-blind, placebo-controlled trial. J. Cardiovasc. Thorac. Res..

[34] Xu D., Feng M., Chu Y., Wang S., Shete V., Tuohy K.M., Liu F., Zhou X., Kamil A., Pan D. (2021). The Prebiotic Effects of Oats on Blood Lipids, Gut Microbiota, and Short-Chain Fatty Acids in Mildly Hypercholesterolemic Subjects Compared With Rice: A Randomized, Controlled Trial. Front. Immunol..

[35] Mezhibovsky E., Wu Y., Bawagan F.G., Tveter K.M., Szeto S., Roopchand D. (2022). Impact of grape polyphenols on *Akkermansia muciniphila* and the gut barrier. AIMS Microbiol..

[36] Roopchand D.E., Carmody R.N., Kuhn P., Moskal K., Rojas-Silva P., Turnbaugh P.J., Raskin I. (2015). Dietary Polyphenols Promote Growth of the Gut Bacterium *Akkermansia muciniphila* and Attenuate High-Fat Diet-Induced Metabolic Syndrome. Diabetes.

[37] Zhu Y., Zhang J.Y., Wei Y.L., Hao J.Y., Lei Y.Q., Zhao W.B., Xiao Y.H., Sun A.D. (2020). The polyphenol-rich extract from chokeberry (*Aronia melanocarpa* L.) modulates gut microbiota and improves lipid metabolism in diet-induced obese rats. Nutr. Metab..

[38] Ramirez J., Guarner F., Bustos Fernandez L., Maruy A., Sdepanian V.L., Cohen H. (2020). Antibiotics as Major Disruptors of Gut Microbiota. Front. Cell. Infect. Microbiol..

[39] Maier L., Goemans C.V., Wirbel J., Kuhn M., Eberl C., Pruteanu M., Muller P., Garcia-Santamarina S., Cacace E., Zhang B. (2021). Unravelling the collateral damage of antibiotics on gut bacteria. Nature.

[40] Ouwerkerk J.P., Tytgat H.L.P., Elzinga J., Koehorst J., Van den Abbeele P., Henrissat B., Gueimonde M., Cani P.D., Van de Wiele T., Belzer C. (2022). Comparative Genomics and Physiology of *Akkermansia muciniphila* Isolates from Human Intestine Reveal Specialized Mucosal Adaptation. Microorganisms.

[41] Dubourg G., Lagier J.C., Armougom F., Robert C., Audoly G., Papazian L., Raoult D. (2013). High-level colonisation of the human gut by Verrucomicrobia following broad-spectrum antibiotic treatment. Int. J. Antimicrob. Agents.

[42] Luo Y., Lan C., Li H., Ouyang Q., Kong F., Wu A., Ren Z., Tian G., Cai J., Yu B. (2022). Rational consideration of *Akkermansia muciniphila* targeting intestinal health: Advantages and challenges. NPJ Biofilms Microbiomes.

[43] Ke H., Li F., Deng W., Li Z., Wang S., Lv P., Chen Y. (2021). Metformin Exerts Anti-inflammatory and Mucus Barrier Protective Effects by Enriching *Akkermansia muciniphila* in Mice With Ulcerative Colitis. Front. Pharmacol..

[44] Zhu X., Shen J., Feng S., Huang C., Wang H., Huo F., Liu H. (2023). *Akkermansia muciniphila*, which is enriched in the gut microbiota by metformin, improves cognitive function in aged mice by reducing the proinflammatory cytokine interleukin-6. Microbiome.

[45] Lee H., Ko G. (2014). Effect of metformin on metabolic improvement and gut microbiota. Appl. Environ. Microbiol..

[46] Regnier M., Rastelli M., Morissette A., Suriano F., Le Roy T., Pilon G., Delzenne N.M., Marette A., Van Hul M., Cani P.D. (2020). Rhubarb Supplementation Prevents Diet-Induced Obesity and Diabetes in Association with Increased *Akkermansia muciniphila* in Mice. Nutrients.

[47] Wang L., Wu Y., Zhuang L., Chen X., Min H., Song S., Liang Q., Li A.D., Gao Q. (2019). Puerarin prevents high-fat diet-induced obesity by enriching *Akkermansia muciniphila* in the gut microbiota of mice. PLoS ONE.

[48] Fujisaka S., Usui I., Nawaz A., Igarashi Y., Okabe K., Furusawa Y., Watanabe S., Yamamoto S., Sasahara M., Watanabe Y. (2020). Bofutsushosan improves gut barrier function with a bloom of *Akkermansia muciniphila* and improves glucose metabolism in mice with diet-induced obesity. Sci. Rep..

[49] Nishiyama M., Ohtake N., Kaneko A., Tsuchiya N., Imamura S., Iizuka S., Ishizawa S., Nishi A., Yamamoto M., Taketomi A. (2020). Increase of *Akkermansia muciniphila* by a Diet Containing Japanese Traditional Medicine Bofutsushosan in a Mouse Model of Non-Alcoholic Fatty Liver Disease. Nutrients.

[50] Collado M.C., Derrien M., Isolauri E., de Vos W.M., Salminen S. (2007). Intestinal integrity and *Akkermansia muciniphila*, a mucin-degrading member of the intestinal microbiota present in infants, adults, and the elderly. Appl. Environ. Microbiol..

[51] Barcena C., Valdes-Mas R., Mayoral P., Garabaya C., Durand S., Rodriguez F., Fernandez-Garcia M.T., Salazar N., Nogacka A.M., Garatachea N. (2019). Healthspan and lifespan extension by fecal microbiota transplantation into progeroid mice. Nat. Med..

[52] van der Lugt B., van Beek A.A., Aalvink S., Meijer B., Sovran B., Vermeij W.P., Brandt R.M.C., de Vos W.M., Savelkoul H.F.J., Steegenga W.T. (2019). *Akkermansia muciniphila* ameliorates the age-related decline in colonic mucus thickness and attenuates immune activation in accelerated aging Ercc1 (-/Delta7) mice. Immun. Ageing.

[53] Kraal L., Abubucker S., Kota K., Fischbach M.A., Mitreva M. (2014). The prevalence of species and strains in the human microbiome: A resource for experimental efforts. PLoS ONE.

[54] Yang J.C., Lagishetty V., Aja E., Arias-Jayo N., Chang C., Hauer M., Katzka W., Zhou Y., Sedighian F., Koletic C. (2024). Biogeographical distribution of gut microbiome composition and function is partially recapitulated by fecal transplantation into germ-free mice. ISME J..

[55] Jacobs J.P., Goudarzi M., Lagishetty V., Li D., Mak T., Tong M., Ruegger P., Haritunians T., Landers C., Fleshner P. (2022). Crohn’s disease in endoscopic remission, obesity, and cases of high genetic risk demonstrates overlapping shifts in the colonic mucosal-luminal interface microbiome. Genome Med..

[56] Derrien M., Vaughan E.E., Plugge C.M., de Vos W.M. (2004). *Akkermansia muciniphila* gen. nov., sp. nov., a human intestinal mucin-degrading bacterium. Int. J. Syst. Evol. Microbiol..

[57] Chia L.W., Hornung B.V.H., Aalvink S., Schaap P.J., de Vos W.M., Knol J., Belzer C. (2018). Deciphering the trophic interaction between *Akkermansia muciniphila* and the butyrogenic gut commensal *Anaerostipes caccae* using a metatranscriptomic approach. Antonie Van Leeuwenhoek.

[58] Shuoker B., Pichler M.J., Jin C., Sakanaka H., Wu H., Gascuena A.M., Liu J., Nielsen T.S., Holgersson J., Nordberg Karlsson E. (2023). Sialidases and fucosidases of *Akkermansia muciniphila* are crucial for growth on mucin and nutrient sharing with mucus-associated gut bacteria. Nat. Commun..

[59] Guo X., Zhang J., Wu F., Zhang M., Yi M., Peng Y. (2016). Different subtype strains of *Akkermansia muciniphila* abundantly colonize in southern China. J. Appl. Microbiol..

[60] Derrien M., Collado M.C., Ben-Amor K., Salminen S., de Vos W.M. (2008). The Mucin degrader *Akkermansia muciniphila* is an abundant resident of the human intestinal tract. Appl. Environ. Microbiol..

[61] Glover J.S., Ticer T.D., Engevik M.A. (2022). Characterizing the mucin-degrading capacity of the human gut microbiota. Sci. Rep..

[62] Ashrafian F., Shahriary A., Behrouzi A., Moradi H.R., Keshavarz Azizi Raftar S., Lari A., Hadifar S., Yaghoubfar R., Ahmadi Badi S., Khatami S. (2019). *Akkermansia muciniphila*-Derived Extracellular Vesicles as a Mucosal Delivery Vector for Amelioration of Obesity in Mice. Front. Microbiol..

[63] Schneeberger M., Everard A., Gomez-Valades A.G., Matamoros S., Ramirez S., Delzenne N.M., Gomis R., Claret M., Cani P.D. (2015). *Akkermansia muciniphila* inversely correlates with the onset of inflammation, altered adipose tissue metabolism and metabolic disorders during obesity in mice. Sci. Rep..

[64] Zhou Q., Pang G., Zhang Z., Yuan H., Chen C., Zhang N., Yang Z., Sun L. (2021). Association Between Gut *Akkermansia* and Metabolic Syndrome is Dose-Dependent and Affected by Microbial Interactions: A Cross-Sectional Study. Diabetes Metab. Syndr. Obes..

[65] He X., Bai Y., Zhou H., Wu K. (2022). *Akkermansia muciniphila* Alters Gut Microbiota and Immune System to Improve Cardiovascular Diseases in Murine Model. Front. Microbiol..

[66] Plovier H., Everard A., Druart C., Depommier C., Van Hul M., Geurts L., Chilloux J., Ottman N., Duparc T., Lichtenstein L. (2017). A purified membrane protein from *Akkermansia muciniphila* or the pasteurized bacterium improves metabolism in obese and diabetic mice. Nat. Med..

[67] Shin N.R., Lee J.C., Lee H.Y., Kim M.S., Whon T.W., Lee M.S., Bae J.W. (2014). An increase in the *Akkermansia* spp. population induced by metformin treatment improves glucose homeostasis in diet-induced obese mice. Gut.

[68] Keane J.M., Las Heras V., Pinheiro J., FitzGerald J.A., Nunez-Sanchez M.A., Hueston C.M., O’Mahony L., Cotter P.D., Hill C., Melgar S. (2023). *Akkermansia muciniphila* reduces susceptibility to Listeria monocytogenes infection in mice fed a high-fat diet. Gut Microbes.

[69] Kleessen B., Hartmann L., Blaut M. (2003). Fructans in the diet cause alterations of intestinal mucosal architecture, released mucins and mucosa-associated bifidobacteria in gnotobiotic rats. Br. J. Nutr..

[70] Yan S., Chen L., Li N., Wei X., Wang J., Dong W., Wang Y., Shi J., Ding X., Peng Y. (2024). Effect of *Akkermansia muciniphila* on pancreatic islet beta-cell function in rats with prediabetes mellitus induced by a high-fat diet. Bioresour. Bioprocess..

[71] Ashammakhi N., Nasiri R., Barros N.R., Tebon P., Thakor J., Goudie M., Shamloo A., Martin M.G., Khademhosseini A. (2020). Gut-on-a-chip: Current progress and future opportunities. Biomaterials.

[72] Rodrigues V.F., Elias-Oliveira J., Pereira I.S., Pereira J.A., Barbosa S.C., Machado M.S.G., Carlos D. (2022). *Akkermansia muciniphila* and Gut Immune System: A Good Friendship That Attenuates Inflammatory Bowel Disease, Obesity, and Diabetes. Front. Immunol..

[73] Mo C., Lou X., Xue J., Shi Z., Zhao Y., Wang F., Chen G. (2024). The influence of *Akkermansia muciniphila* on intestinal barrier function. Gut Pathog..

[74] Ottman N., Huuskonen L., Reunanen J., Boeren S., Klievink J., Smidt H., Belzer C., de Vos W.M. (2016). Characterization of Outer Membrane Proteome of *Akkermansia muciniphila* Reveals Sets of Novel Proteins Exposed to the Human Intestine. Front. Microbiol..

[75] Qu D., Chen M., Zhu H., Liu X., Cui Y., Zhou W., Zhang M. (2023). *Akkermansia muciniphila* and its outer membrane protein Amuc_1100 prevent high-fat diet-induced nonalcoholic fatty liver disease in mice. Biochem. Biophys. Res. Commun..

[76] Li J., Lin S., Vanhoutte P.M., Woo C.W., Xu A. (2016). *Akkermansia muciniphila* Protects Against Atherosclerosis by Preventing Metabolic Endotoxemia-Induced Inflammation in Apoe-/- Mice. Circulation.

[77] Zhai Q., Feng S., Arjan N., Chen W. (2019). A next generation probiotic, *Akkermansia muciniphila*. Crit. Rev. Food Sci. Nutr..

[78] Effendi R., Anshory M., Kalim H., Dwiyana R.F., Suwarsa O., Pardo L.M., Nijsten T.E.C., Thio H.B. (2022). *Akkermansia muciniphila* and Faecalibacterium prausnitzii in Immune-Related Diseases. Microorganisms.

[79] Zheng M., Han R., Yuan Y., Xing Y., Zhang W., Sun Z., Liu Y., Li J., Mao T. (2022). The role of *Akkermansia muciniphila* in inflammatory bowel disease: Current knowledge and perspectives. Front. Immunol..

[80] He K.Y., Lei X.Y., Wu D.H., Zhang L., Li J.Q., Li Q.T., Yin W.T., Zhao Z.L., Liu H., Xiang X.Y. (2023). *Akkermansia muciniphila* protects the intestine from irradiation-induced injury by secretion of propionic acid. Gut Microbes.

[81] Ansaldo E., Slayden L.C., Ching K.L., Koch M.A., Wolf N.K., Plichta D.R., Brown E.M., Graham D.B., Xavier R.J., Moon J.J. (2019). *Akkermansia muciniphila* induces intestinal adaptive immune responses during homeostasis. Science.

[82] Liu Y., Yang M., Tang L., Wang F., Huang S., Liu S., Lei Y., Wang S., Xie Z., Wang W. (2022). TLR4 regulates RORgammat(+) regulatory T-cell responses and susceptibility to colon inflammation through interaction with *Akkermansia muciniphila*. Microbiome.

[83] Kimura I., Ichimura A., Ohue-Kitano R., Igarashi M. (2020). Free Fatty Acid Receptors in Health and Disease. Physiol. Rev..

[84] Vinolo M.A., Rodrigues H.G., Hatanaka E., Hebeda C.B., Farsky S.H., Curi R. (2009). Short-chain fatty acids stimulate the migration of neutrophils to inflammatory sites. Clin. Sci..

[85] Macia L., Tan J., Vieira A.T., Leach K., Stanley D., Luong S., Maruya M., Ian McKenzie C., Hijikata A., Wong C. (2015). Metabolite-sensing receptors GPR43 and GPR109A facilitate dietary fibre-induced gut homeostasis through regulation of the inflammasome. Nat. Commun..

[86] Wu Z.Q., Chen X.M., Ma H.Q., Li K., Wang Y.L., Li Z.J. (2023). *Akkermansia muciniphila* Cell-Free Supernatant Improves Glucose and Lipid Metabolisms in Caenorhabditis elegans. Nutrients.

[87] Shen J., Tong X., Sud N., Khound R., Song Y., Maldonado-Gomez M.X., Walter J., Su Q. (2016). Low-Density Lipoprotein Receptor Signaling Mediates the Triglyceride-Lowering Action of *Akkermansia muciniphila* in Genetic-Induced Hyperlipidemia. Arterioscler. Thromb. Vasc. Biol..

[88] Yang M., Bose S., Lim S., Seo J., Shin J., Lee D., Chung W.H., Song E.J., Nam Y.D., Kim H. (2020). Beneficial Effects of Newly Isolated *Akkermansia muciniphila* Strains from the Human Gut on Obesity and Metabolic Dysregulation. Microorganisms.

[89] Xia J., Lv L., Liu B., Wang S., Zhang S., Wu Z., Yang L., Bian X., Wang Q., Wang K. (2022). *Akkermansia muciniphila* Ameliorates Acetaminophen-Induced Liver Injury by Regulating Gut Microbial Composition and Metabolism. Microbiol. Spectr..

[90] Zheng X., Huang W., Li Q., Chen Y., Wu L., Dong Y., Huang X., He X., Ou Z., Peng Y. (2023). Membrane Protein Amuc_1100 Derived from *Akkermansia muciniphila* Facilitates Lipolysis and Browning via Activating the AC3/PKA/HSL Pathway. Microbiol. Spectr..

[91] Ashrafian F., Keshavarz Azizi Raftar S., Lari A., Shahryari A., Abdollahiyan S., Moradi H.R., Masoumi M., Davari M., Khatami S., Omrani M.D. (2021). Extracellular vesicles and pasteurized cells derived from *Akkermansia muciniphila* protect against high-fat induced obesity in mice. Microb. Cell Fact..

[92] Yoon H.S., Cho C.H., Yun M.S., Jang S.J., You H.J., Kim J.H., Han D., Cha K.H., Moon S.H., Lee K. (2021). *Akkermansia muciniphila* secretes a glucagon-like peptide-1-inducing protein that improves glucose homeostasis and ameliorates metabolic disease in mice. Nat. Microbiol..

[93] Depommier C., Van Hul M., Everard A., Delzenne N.M., De Vos W.M., Cani P.D. (2020). Pasteurized *Akkermansia muciniphila* increases whole-body energy expenditure and fecal energy excretion in diet-induced obese mice. Gut Microbes.

[94] Lopetuso L.R., Quagliariello A., Schiavoni M., Petito V., Russo A., Reddel S., Del Chierico F., Ianiro G., Scaldaferri F., Neri M. (2020). Towards a disease-associated common trait of gut microbiota dysbiosis: The pivotal role of *Akkermansia muciniphila*. Dig. Liver Dis..

[95] Wu W., Kaicen W., Bian X., Yang L., Ding S., Li Y., Li S., Zhuge A., Li L. (2023). *Akkermansia muciniphila* alleviates high-fat-diet-related metabolic-associated fatty liver disease by modulating gut microbiota and bile acids. Microb. Biotechnol..

[96] Fang C., Cheng J., Jia W., Xu Y. (2023). *Akkermansia muciniphila* Ameliorates Alcoholic Liver Disease in Experimental Mice by Regulating Serum Metabolism and Improving Gut Dysbiosis. Metabolites.

[97] Keshavarz Azizi Raftar S., Ashrafian F., Yadegar A., Lari A., Moradi H.R., Shahriary A., Azimirad M., Alavifard H., Mohsenifar Z., Davari M. (2021). The Protective Effects of Live and Pasteurized *Akkermansia muciniphila* and Its Extracellular Vesicles against HFD/CCl4-Induced Liver Injury. Microbiol. Spectr..

[98] Guo M., Lu M., Chen K., Xu R., Xia Y., Liu X., Liu Z., Liu Q. (2023). *Akkermansia muciniphila* and Lactobacillus plantarum ameliorate systemic lupus erythematosus by possibly regulating immune response and remodeling gut microbiota. mSphere.

[99] Wang B., Chen X., Chen Z., Xiao H., Dong J., Li Y., Zeng X., Liu J., Wan G., Fan S. (2023). Stable colonization of *Akkermansia muciniphila* educates host intestinal microecology and immunity to battle against inflammatory intestinal diseases. Exp. Mol. Med..

[100] Xu Y., Duan J., Wang D., Liu J., Chen X., Qin X.Y., Yu W. (2023). *Akkermansia muciniphila* Alleviates Persistent Inflammation, Immunosuppression, and Catabolism Syndrome in Mice. Metabolites.

[101] Song B., Xian W., Sun Y., Gou L., Guo Q., Zhou X., Ren B., Cheng L. (2023). *Akkermansia muciniphila* inhibited the periodontitis caused by *Fusobacterium nucleatum*. NPJ Biofilms Microbiomes.

[102] Letchumanan G., Abdullah N., Marlini M., Baharom N., Lawley B., Omar M.R., Mohideen F.B.S., Addnan F.H., Nur Fariha M.M., Ismail Z. (2022). Gut Microbiota Composition in Prediabetes and Newly Diagnosed Type 2 Diabetes: A Systematic Review of Observational Studies. Front. Cell. Infect. Microbiol..

[103] Depommier C., Everard A., Druart C., Plovier H., Van Hul M., Vieira-Silva S., Falony G., Raes J., Maiter D., Delzenne N.M. (2019). Supplementation with *Akkermansia muciniphila* in overweight and obese human volunteers: A proof-of-concept exploratory study. Nat. Med..

[104] Osman M.A., Neoh H.M., Ab Mutalib N.S., Chin S.F., Mazlan L., Raja Ali R.A., Zakaria A.D., Ngiu C.S., Ang M.Y., Jamal R. (2021). *Parvimonas micra*, *Peptostreptococcus stomatis*, *Fusobacterium nucleatum* and *Akkermansia muciniphila* as a four-bacteria biomarker panel of colorectal cancer. Sci. Rep..

[105] Png C.W., Linden S.K., Gilshenan K.S., Zoetendal E.G., McSweeney C.S., Sly L.I., McGuckin M.A., Florin T.H. (2010). Mucolytic bacteria with increased prevalence in IBD mucosa augment in vitro utilization of mucin by other bacteria. Am. J. Gastroenterol..

[106] Depommier C., Everard A., Druart C., Maiter D., Thissen J.P., Loumaye A., Hermans M.P., Delzenne N.M., de Vos W.M., Cani P.D. (2021). Serum metabolite profiling yields insights into health promoting effect of *A. muciniphila* in human volunteers with a metabolic syndrome. Gut Microbes.

[107] Cruz-Aguliar R.M., Wantia N., Clavel T., Vehreschild M., Buch T., Bajbouj M., Haller D., Busch D., Schmid R.M., Stein-Thoeringer C.K. (2019). An Open-Labeled Study on Fecal Microbiota Transfer in Irritable Bowel Syndrome Patients Reveals Improvement in Abdominal Pain Associated with the Relative Abundance of *Akkermansia muciniphila*. Digestion.

[108] Herrera-deGuise C., Varela E., Sarrabayrouse G., Pozuelo Del Rio M., Alonso V.R., Sainz N.B., Casellas F., Mayorga L.F., Manichanh C., Vidaur F.A. (2023). Gut Microbiota Composition in Long-Remission Ulcerative Colitis is Close to a Healthy Gut Microbiota. Inflamm. Bowel Dis..

[109] Salgia N.J., Bergerot P.G., Maia M.C., Dizman N., Hsu J., Gillece J.D., Folkerts M., Reining L., Trent J., Highlander S.K. (2020). Stool Microbiome Profiling of Patients with Metastatic Renal Cell Carcinoma Receiving Anti-PD-1 Immune Checkpoint Inhibitors. Eur. Urol..

[110] Dao M.C., Everard A., Aron-Wisnewsky J., Sokolovska N., Prifti E., Verger E.O., Kayser B.D., Levenez F., Chilloux J., Hoyles L. (2016). *Akkermansia muciniphila* and improved metabolic health during a dietary intervention in obesity: Relationship with gut microbiome richness and ecology. Gut.

[111] Lei W., Cheng Y., Gao J., Liu X., Shao L., Kong Q., Zheng N., Ling Z., Hu W. (2023). *Akkermansia muciniphila* in neuropsychiatric disorders: Friend or foe?. Front. Cell. Infect. Microbiol..

[112] Zhang H., DiBaise J.K., Zuccolo A., Kudrna D., Braidotti M., Yu Y., Parameswaran P., Crowell M.D., Wing R., Rittmann B.E. (2009). Human gut microbiota in obesity and after gastric bypass. Proc. Natl. Acad. Sci. USA.

[113] He K., Hu Y., Ma H., Zou Z., Xiao Y., Yang Y., Feng M., Li X., Ye X. (2016). Rhizoma Coptidis alkaloids alleviate hyperlipidemia in B6 mice by modulating gut microbiota and bile acid pathways. Biochim. Biophys. Acta.

[114] Kim S., Lee Y., Kim Y., Seo Y., Lee H., Ha J., Lee J., Choi Y., Oh H., Yoon Y. (2020). *Akkermansia muciniphila* Prevents Fatty Liver Disease, Decreases Serum Triglycerides, and Maintains Gut Homeostasis. Appl. Environ. Microbiol..

[115] Hasani A., Ebrahimzadeh S., Hemmati F., Khabbaz A., Hasani A., Gholizadeh P. (2021). The role of *Akkermansia muciniphila* in obesity, diabetes and atherosclerosis. J. Med. Microbiol..

[116] Liou A.P., Paziuk M., Luevano J.M., Machineni S., Turnbaugh P.J., Kaplan L.M. (2013). Conserved shifts in the gut microbiota due to gastric bypass reduce host weight and adiposity. Sci. Transl. Med..

[117] Dong T.S., Katzka W., Yang J.C., Chang C., Arias-Jayo N., Lagishetty V., Balioukova A., Chen Y., Dutson E., Li Z. (2023). Microbial changes from bariatric surgery alters glucose-dependent insulinotropic polypeptide and prevents fatty liver disease. Gut Microbes.

[118] Khalili L., Centner A.M., Salazar G. (2023). Effects of Berries, Phytochemicals, and Probiotics on Atherosclerosis through Gut Microbiota Modification: A Meta-Analysis of Animal Studies. Int. J. Mol. Sci..

[119] Han B., Chao K., Wang D., Sun Y., Ding X., Zhang X., Liu S., Du J., Luo Y., Wang H. (2023). A purified membrane protein from *Akkermansia muciniphila* blunted the sepsis-induced acute lung injury by modulation of gut microbiota in rats. Int. Immunopharmacol..

[120] Lee Y., Byeon H.R., Jang S.Y., Hong M.G., Kim D., Lee D., Shin J.H., Kim Y., Kang S.G., Seo J.G. (2022). Oral administration of Faecalibacterium prausnitzii and *Akkermansia muciniphila* strains from humans improves atopic dermatitis symptoms in DNCB induced NC/Nga mice. Sci. Rep..

[121] Wang L., Tang L., Feng Y., Zhao S., Han M., Zhang C., Yuan G., Zhu J., Cao S., Wu Q. (2020). A purified membrane protein from *Akkermansia muciniphila* or the pasteurised bacterium blunts colitis associated tumourigenesis by modulation of CD8(+) T cells in mice. Gut.

[122] Zhu Z., Cai J., Hou W., Xu K., Wu X., Song Y., Bai C., Mo Y.Y., Zhang Z. (2023). Microbiome and spatially resolved metabolomics analysis reveal the anticancer role of gut *Akkermansia muciniphila* by crosstalk with intratumoral microbiota and reprogramming tumoral metabolism in mice. Gut Microbes.

[123] Souza R.O., Miranda V.C., Quintanilha M.F., Gallotti B., Oliveira S.R.M., Silva J.L., Alvarez-Leite J.I., Jesus L.C.L., Azevedo V., Vital K.D. (2024). Evaluation of the Treatment with *Akkermansia muciniphila* BAA-835 of Chemotherapy-induced Mucositis in Mice. Probiotics Antimicrob. Proteins.

[124] Vakili B., Fateh A., Asadzadeh Aghdaei H., Sotoodehnejadnematalahi F., Siadat S.D. (2020). Intestinal Microbiota in Elderly Inpatients with Clostridioides difficile Infection. Infect. Drug Resist..

[125] Vakili B., Fateh A., Asadzadeh Aghdaei H., Sotoodehnejadnematalahi F., Siadat S.D. (2020). Characterization of Gut Microbiota in Hospitalized Patients with Clostridioides difficile Infection. Curr. Microbiol..

[126] Wu Z., Xu Q., Gu S., Chen Y., Lv L., Zheng B., Wang Q., Wang K., Wang S., Xia J. (2022). *Akkermansia muciniphila* Ameliorates Clostridioides difficile Infection in Mice by Modulating the Intestinal Microbiome and Metabolites. Front. Microbiol..

[127] Shang Q., Wang Y., Pan L., Niu Q., Li C., Jiang H., Cai C., Hao J., Li G., Yu G. (2018). Dietary Polysaccharide from Enteromorpha Clathrata Modulates Gut Microbiota and Promotes the Growth of *Akkermansia muciniphila*, *Bifidobacterium* spp. and *Lactobacillus* spp.. Mar. Drugs.

[128] Muchhala K.H., Kallurkar P.S., Kang M., Koseli E., Poklis J.L., Xu Q., Dewey W.L., Fettweis J.M., Jimenez N.R., Akbarali H.I. (2024). The role of morphine- and fentanyl-induced impairment of intestinal epithelial antibacterial activity in dysbiosis and its impact on the microbiota-gut-brain axis. FASEB J..

[129] Sanjiwani M.I.D., Aryadi I.P.H., Semadi I.M.S. (2022). Review of Literature on *Akkermansia muciniphila* and its Possible Role in the Etiopathogenesis and Therapy of Type 2 Diabetes Mellitus. J. ASEAN Fed. Endocr. Soc..

[130] Ma J., Liu Z., Gao X., Bao Y., Hong Y., He X., Zhu W., Li Y., Huang W., Zheng N. (2023). Gut microbiota remodeling improves natural aging-related disorders through *Akkermansia muciniphila* and its derived acetic acid. Pharmacol. Res..

[131] Jiang Y., Xu Y., Zheng C., Ye L., Jiang P., Malik S., Xu G., Zhou Q., Zhang M. (2023). Acetyltransferase from *Akkermansia muciniphila* blunts colorectal tumourigenesis by reprogramming tumour microenvironment. Gut.

[132] Xie S., Li J., Lyu F., Xiong Q., Gu P., Chen Y., Chen M., Bao J., Zhang X., Wei R. (2023). Novel tripeptide RKH derived from *Akkermansia muciniphila* protects against lethal sepsis. Gut.

[133] Chelakkot C., Choi Y., Kim D.K., Park H.T., Ghim J., Kwon Y., Jeon J., Kim M.S., Jee Y.K., Gho Y.S. (2018). *Akkermansia muciniphila*-derived extracellular vesicles influence gut permeability through the regulation of tight junctions. Exp. Mol. Med..

[134] Perraudeau F., McMurdie P., Bullard J., Cheng A., Cutcliffe C., Deo A., Eid J., Gines J., Iyer M., Justice N. (2020). Improvements to postprandial glucose control in subjects with type 2 diabetes: A multicenter, double blind, randomized placebo-controlled trial of a novel probiotic formulation. BMJ Open Diabetes Res. Care.

[135] Kang C.H., Jung E.S., Jung S.J., Han Y.H., Chae S.W., Jeong D.Y., Kim B.C., Lee S.O., Yoon S.J. (2024). Pasteurized *Akkermansia muciniphila* HB05 (HB05P) Improves Muscle Strength and Function: A 12-Week, Randomized, Double-Blind, Placebo-Controlled Clinical Trial. Nutrients.

[136] Lee H.W., Lee S.N., Seo J.G., Koo Y., Kang S.Y., Choi C.W., Park S.Y., Lee S.Y., Kim S.R., Kim J.H. (2024). Efficacy of ETB-F01, Heat-Killed *Akkermansia muciniphila* Strain EB-AMDK19, in Patients with Respiratory Symptoms: A Multicenter Clinical Trial. Nutrients.

[137] Dehghanbanadaki H., Aazami H., Keshavarz Azizi Raftar S., Ashrafian F., Ejtahed H.S., Hashemi E., Hoseini Tavassol Z., Ahmadi Badi S., Siadat S.D. (2020). Global scientific output trend for *Akkermansia muciniphila* research: A bibliometric and scientometric analysis. BMC Med. Inform. Decis. Mak..

[138] Earley H., Lennon G., Balfe A., Coffey J.C., Winter D.C., O’Connell P.R. (2019). The abundance of *Akkermansia muciniphila* and its relationship with sulphated colonic mucins in health and ulcerative colitis. Sci. Rep..

[139] Karcher N., Nigro E., Puncochar M., Blanco-Miguez A., Ciciani M., Manghi P., Zolfo M., Cumbo F., Manara S., Golzato D. (2021). Genomic diversity and ecology of human-associated *Akkermansia* species in the gut microbiome revealed by extensive metagenomic assembly. Genome Biol..

